# Posterior urethral valve in thai boys

**DOI:** 10.1186/s12887-023-04281-x

**Published:** 2023-09-07

**Authors:** Prakorn Ekarat, Worapat Attawettayanon, Chompoonut Limratchapong, Praewa Sophark, Prayong Vachvanichsanong

**Affiliations:** 1 Present Address: Department of Pediatrics, Faculty of Medicine, Prince of Songkla University, Hat Yai, Songkhla 90110 Thailand; 2 Present Address: Department of Surgery, Faculty of Medicine, Prince of Songkla University, Hat Yai, Songkhla, 90110 Thailand

**Keywords:** Acute kidney injury, Chronic kidney disease, Hydronephrosis, Posterior urethral valve, Urinary tract infection, Urinary tract obstruction, Vesicoureteral reflux

## Abstract

**Background:**

Posterior urethral valve (PUV) is the most common congenital bladder outlet obstruction in boys, causing renal damage beginning in utero. There are scarce data from Thailand regarding the long-term outcomes of PUV in boys, thus the aim of this study was to examine the presentation, clinical course, complications, outcomes and renal survival in PUV boys.

**Methods:**

We reviewed the medical records of PUV boys treated at the Pediatric Nephrology Clinic, Prince of Songkla University, Thailand, over a 30-year-period.

**Results:**

Seventy-seven PUV boys were identified, with a median age at diagnosis of 4.8 months. The most common presentations were urinary tract infection (UTI), poor urine stream and urinary dribbling in 26 (33.8%), 19 (24.7%) and 11 (14.3%) boys, respectively. Renal ultrasound results in 70 boys showed 8 (11.4%) unilateral and 56 (80%) bilateral hydronephroses. Of 72 voiding cystourethrograms, 18 (25.0%) showed unilateral and 22 (30.6%) bilateral vesicoureteral refluxes. ^99m^Tc dimercaptosuccinic acid renal scans in 30 boys showed 12 (40%) unilateral and 8 (26.7%) bilateral renal damage. Fifty-nine (76.6%) boys had 149 UTIs; 42 (54.4%) had recurrent UTI. Forty-eight boys had valve ablation at the median age of 30.3 months. 22 boys (28.6%) developed chronic kidney disease (CKD) at a median age of 15.0 years.

**Conclusion:**

Of 77 PUV Thai boys, UTI was the most common presentation. Recurrence of UTI and CKD was the most common consequence. Lifelong follow-up for renal and bladder functions is essential for all PUV patients.

## Introduction

Posterior urethral valve (PUV) is a congenital anomaly of the prostatic urethra in boys in the presence of a mucosal membrane causing urinary bladder outlet obstruction [1]. PUV is the most common cause of urinary outlet obstruction leading to renal damage. Additionally, PUV is a major cause of chronic kidney disease (CKD) in infant boys [2, 3]. The incidence of PUV has been reported at 1:5,000–1:8,000 males [4].

The prevalences of CKD and eventually lifetime end-stage renal disease (ESRD) following PUV varies depending on the severity of disease, age at diagnosis, and follow up period, and have been reported at 20–30% [5–8]. The consequences of PUV as the result of urinary retention include high morbidity (e.g. acute kidney injury (AKI), urinary tract infections (UTI), urosepsis, urinary overflow incontinence, poor quality of life) and high mortality due to CKD and sequelae (e.g. hypertension, anemia, failure to thrive, osteodystrophy, and renal replacement therapy problems) [5, 9, 10]. One study reported 23 of 67 (34.8%) Iranian PUV boys developed ESRD at the ages of 1–15 years, with a mean renal survival rate of 7.8 years [11].

### Objective

To determine the presentation, clinical course, therapy and outcomes of a cohort of Thai boys with PUV in a major tertiary care center, and to see if there were any reliable predictor variables of CKD development.

### Patients and methods

We retrospectively reviewed the medical records of all boys diagnosed as PUV treated at the Pediatric Nephrology Clinic, Department of Pediatrics, Songklanagarind Hospital, Prince of Songkla University, Thailand during 1991–2020.

### Definitions

PUV was diagnosed based on a voiding cystourethrogram showing a valve leaflet and dilated and elongated posterior urethra [1]. Bladder dysfunction was diagnosed by a history of urinary dribbling and significant residual urine. Hypertension, UTI, proteinuria, AKI, CKD, hydronephrosis, vesicoureteral reflux (VUR), renal scarring and dysplasia were based on standard definitions [12–19]. Failure to thrive was defined based on normal references of the Public Health Ministry, Thailand for age and gender.

### Statistical analysis

Descriptive results are presented as means and standard deviations or medians and interquartile ranges (IQR) for continuous variables as appropriate and frequencies and percentages for categorical variables. Fisher’s exact test, rank-sum test or chi-square tests were performed to compare categorical factors between children with/without CKD.Statistical significance was considered as a p-value < 0.05. R version 3.6.0 was used for all analyses [20].

## Results

**Fig. 1:**
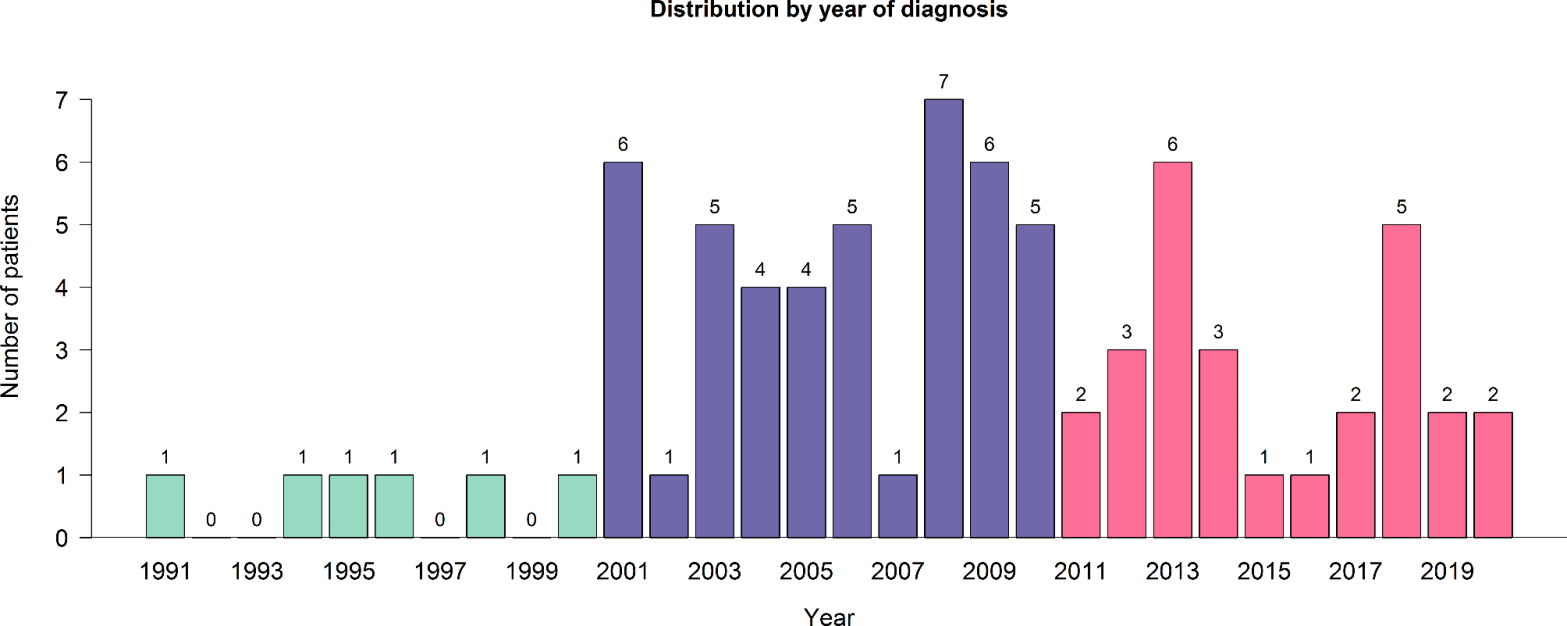
Number of boys with posterior urethral valves in this study by year

A total of 82 medical records of boys diagnosed as PUV during the 30-year study period were reviewed, with 5 excluded for missing data. The 77 remaining boys were diagnosed at a median age of 4.8 months (IQR 0.8–28.7). The years of diagnosis, presentations, ages at last visit, UTI episodes, and outcomes at follow up including CKD development by age distributions are shown in Fig. 1; Table 1.

**Table 1:** Distribution of 77 study posterior urethral valve boys by age group and period of diagnosis, presentation, urinary tract infection, chronic kidney disease and last status

Age group	0–3 mo	> 3–6 mo	> 6–12 mo	> 12–36 mo	> 36–60 mo	> 60 mo	Total
**Total n (%)**	32	13	7	7	10	8	77
**Period of diagnosis** 1991–2000 2001–2010 2011–2020	3 (9.4) 17 (53.1) 12 (37.5)	1 (7.7) 7 (53.8) 5 (38.5)	0 4 (57.1) 3 (42.9)	2 (28.6) 3 (42.9) 2 (28.6)	0 7 (70.0) 3 (30.0)	0 6 (75.0) 2 (25.0)	6 (7.8) 44 (57.1) 27 (35.1)
**Presentation** UTI Poor urine stream/urine dribbling AKI Not recorded Abdominal mass Ascites	8 (25.0) 12 (37.5) 6 (18.8) 2 (6.3) 3 (9.4) 1 (3.1)	5 (38.5) 6 (46.2) 0 1 (7.7) 1 (7.7) 0	4 (57.1) 3 (42.9) 0 0 0 0	4 (57.1) 0 1 (14.3) 1 (14.3) 1 (14.3) 0	3 (30.0) 6 (60.0) 1 (10.0) 0 0 0	2 (25.0) 3 (37.5) 1 (12.5) 2 (25.0) 0 0	26 (33.8) 30 (39.0) 8 (10.4) 7 (9.1) 5 (6.5) 1 (1.3)
**Age at last visit (yr)** Median (IQR)	3.7 (0.8–10.2)	5.9 (1.8–11.2)	4.2 (2.9–5.1)	12.6 (8.7–22.2)	6.6 (4.8–12.0)	15.7 (12–20.6)	6.3 (2.2–12.6)
**Chronic kidney disease** Yes No	5 (15.6) 27 (84.4)	4 (30.8) 9 (69.2)	1 (14.3) 6 (85.7)	3 (42.9) 4 (57.1)	4 (40.0) 6 (60.0)	5 (62.5) 3 (37.5)	22 (28.6) 55 (71.4)
**Status at last visit** Alive Dead Referred Lost to follow up	14 (43.8) 5 (15.6) 5 (15.6) 8 (25.0)	7 (53.8) 0 3 (23.1) 3 (23.1)	3 (42.9) 0 1 (14.3) 3 (42.9)	5 (71.4) 0 1 (14.3) 1 (14.3)	3 (30.0) 0 6 (60.0) 1 (10.0)	3 (37.5) 0 2 (25.0) 3 (37.5)	35 (45.5) 5 (6.5) 18 (23.4) 19 (24.7)
**UTI episodes** 0 1 2 >2	4 (12.5) 5 (15.6) 9 (28.1) 14 (43.8)	4 (30.8) 3 (23.1) 2 (15.4) 4 (30.8)	0 3 (42.9) 3 (42.9) 1 (14.3)	1 (14.3) 0 3 (42.9) 3 (42.9)	5 (50.0) 4 (40.0) 1 (10.0) 0	4 (50.0) 2 (25.0) 2 (25.0) 0	18 (23.4) 17 (22.1) 20 (26.0) 22 (28.6)

AKI: acute kidney injury; CKD: chronic kidney disease; UTI: urinary tract infection

### Imaging findings

Renal ultrasound results were available for 70 boys. Hydronephrosis was detected in 64 boys (91.4%) of whom 8 (11.4%) and 56 (80.0%) boys had unilateral and bilateral hydronephrosis, respectively. Mild, moderate and severe hydronephrosis were found in 10 (14.3%), 23 (32.9%) and 31 (44.3%) boys, respectively. Of these 120 hydronephrosis kidneys, 19, 42 and 59 had mild, moderate and severe hydronephrosis, respectively. Hydroureter was found in 30 (42.9%) boys with 1 (1.4%) and 29 (41.4%) unilateral and bilateral, respectively.

72 VCUG results were available for VUR evaluation; VUR was detected in 40 boys (55.6%) of which 18 (25.0%) and 22 (30.6%) were unilateral and bilateral, respectively. VUR grades I-V were found in 6 (8.3%), 2 (2.8%), 3 (4.2%), 8 (11.1%) and 21 (29.2%) boys, respectively. Of these 62 VURs, 8, 2, 6, 14 and 32 kidneys were VUR grades I-V, respectively.

^99m^Tc dimercaptosuccinic acid (DMSA) renal scans were performed in 30 boys; 10 boys were normal while 20 (66.7%) had renal scarring and/or renal dysplasia. Twelve and 8 boys had unilateral and bilateral renal damage, respectively, of which there were 18 scarred and 10 dysplastic kidneys.

### UTI and PUV

Fifty-nine (76.6%) boys experienced a total of 149 UTIs, while 42 (54.4%) boys had recurrent UTI. Overall there were 1–7 UTI episodes in 17, 20, 10, 5, 3, 1 and 3 boys, respectively.

### Surgical interventions

Of the 77 boys, 62 had surgical intervention, 48 valve ablation surgeries at a median age of 30.3 months (IQR 12.8–54.8) (17 without prior surgery, 29 following vesicostomy and 2 following both cystostomy and vesicostomy), 14 who had urinary bladder diversion, 11 vesicostomies, 2 cystostomies and 1 boy had a vesicostomy and later had a cystostomy.

### Kidney survival

**Fig. 2:**
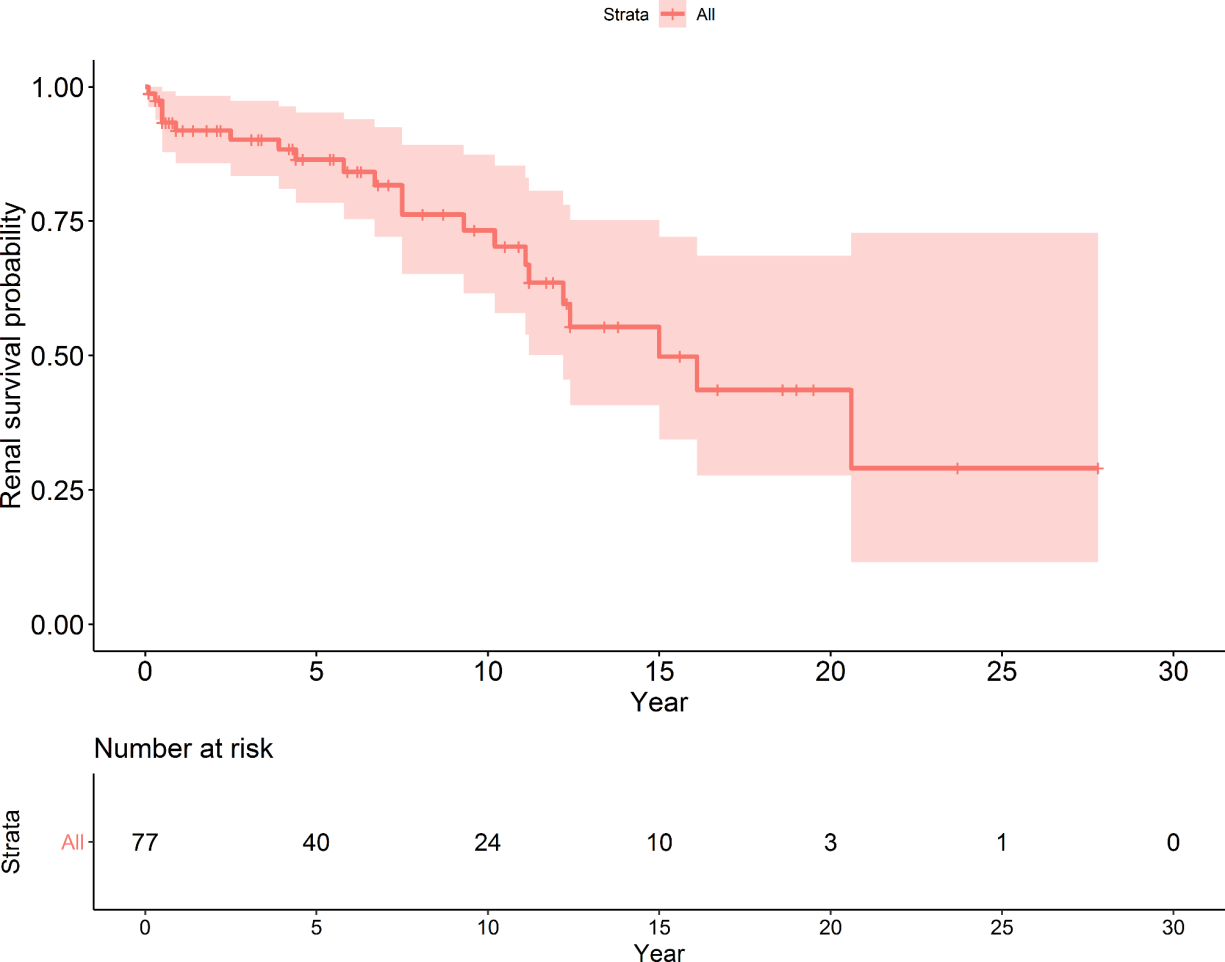
Renal survival curve in 77 study posterior urethral valve boys

Twenty-two of the 77 boys (28.6%) developed CKD at a median age of 15.0 years (IQR 12.2 - ); 2 boys had had CKD since birth. The 1-, 5- and 10-year renal survival rates were 91.8%, 86.4% and 73.3%, respectively. (Fig. 2)

### Patient status

The median age at their last visit was 6.3 years (IQR 2.2–12.6); at that time there were 35 survivors, 5 deceased, and 37 lost to follow up for more than one year after their last visit. 22 boys had had CKD (8 alive, 2 dead, 8 returned to their original hospital and 4 lost to follow up). Of the 8 surviving patients still under our care, 7 went on to ESRD, 2 were on CAPD, 3 on hemodialysis and 2 had had kidney transplantation. The causes of death of the 3 non-CKD patients who had died were pneumonia and severe infection, and one boy had also had multiple organ anomalies.

Table 2 compares the demographic data of the children with and without CKD, and the analysis found no significant variables which could reliably predict CKD. Table 3 compares the imaging results; hydronephrosis, hydroureter VUR and renal damage including age at valve ablation were not significantly different between the boys with/without CKD.

**Table 2:** Comparison of period of diagnosis, age of diagnosis and last visit, UTI episodes, treatment and outcomes in study posterior urethral valve boys with and without chronic kidney disease

	With CKD	Without CKD	Total	Statistical test	P value
**N**	22	55	77		
**Period of diagnosis** 1991–2000 2001–2010 2011–2020	2 (9.1) 16 (72.7) 4 (18.2)	4 (7.3) 28 (50.9) 23 (41.8)	6 (7.8) 44 (57.1) 27 (35.1)	Fisher’s exact test	0.132
**Age at diagnosis** median (IQR) mo	21.5 (3.7–52.2)	3.5 (0.7–11.9)	4.8 (0.8–28.7)	Rank-sum test	*0.013
**Age group at diagnosis** 0–3 mo >3–36 mo >36 mo	5 (22.7) 8 (36.4) 9 (40.9)	27 (49.1) 19 (34.5) 9 (16.4)	32 (41.6) 27 (35.1) 18 (23.4)	Chi sq. (2 df) = 6.69	*0.035
**Maximum creatinine** Median (IQR)	5.2 (2.6–10.2)	0.9 (0.6–1.4)	1.2 (0.6–3.0)	Rank-sum test	< 0.001
**Age at last visit** mean (SD) yr	11.9 (6.1–17.9)	4.6 (2.1–11.1)	6.3 (2.2–12.6)	Rank-sum test	*0.019
**UTI episodes** 0 1 2 >2	5 (22.7) 3 (13.6) 7 (31.8) 7 (31.8)	13 (23.6) 14 (25.5) 13 (23.6) 15 (27.3)	18 (23.4) 17 (22.1) 20 (26) 22 (28.6)	Chi sq. (3 df) = 1.52	0.678
**Status at last visit** Alive Dead Referred Lost to follow up	8 (36.4) 2 (9.1) 8 (36.4) 4 (18.2)	27 (49.1) 3 (5.5) 10 (18.2) 15 (27.3)	35 (45.5) 5 (6.5) 18 (23.4) 19 (24.7)	Fisher’s exact test	0.295
**Failure to thrive** Yes No	10 (45.5) 12 (54.5)	9 (16.4) 46 (83.6)	19 (24.7) 58 (75.3)	Chi sq. (1 df) = 5.68	*0.017
**Proteinuria** Yes No	11 (50) 11 (50)	3 (5.5) 52 (94.5)	14 (18.2) 63 (81.8)	Fisher’s exact test	*< 0.001
**Bladder dysfunction** Yes No	4 (18.2) 18 (81.8)	7 (12.7) 48 (87.3)	11 (14.3) 66 (85.7)	Fisher’s exact test	0.719
**Hypertension** Yes No	7 (31.8) 15 (68.2)	0 55 (100.0)	7 (9.1) 70 (90.9)	Fisher’s exact test	*< 0.001
**Hematuria** Yes No	4 (18.2) 18 (81.8)	0 55 (100.0)	4 (5.2) 73 (94.8)	Fisher’s exact test	*0.005

CKD: chronic kidney disease; IQR: interquartile range; SD: standard deviation; VUR: vesicoureteral reflux

**Table 3:** Comparison of imaging results and age of urethral valve ablation in study posterior urethral valve boys with and without chronic kidney disease

	With CKD	Without CKD	Total	Statistical test	P value
***Renal Ultrasound (N)***	18	52	70		
**Hydronephrosis** No Unilateral Bilateral **Hydroureter** No Unilateral Bilateral	0 2 (11.1) 16 (88.9) 14 (77.8) 0 4 (22.2)	6 (11.5) 6 (11.5) 40 (76.9) 26 (50.0) 1 (1.9) 25 (48.1)	6 (8.6) 8 (11.4) 56 (80.0) 40 (57.1) 1 (1.4) 29 (41.4)	Fisher’s exact test Fisher’s exact test	0.454 0.083
***Voiding cystourethrogram (N)***	19	53	72		
**VUR** No Unilateral VUR Bilateral VUR	8 (42.1) 4 (21.1) 7 (36.8)	24 (45.3) 14 (26.4) 15 (28.3)	32 (44.4) 18 (25) 22 (30.6)	Chi sq. (2 df) = 0.53	0.769
***DMSA Scan (N)***	5	25	30		
**Renal damage** No Unilateral Bilateral	1 (20.0) 3 (60.0) 1 (20.0)	9 (36.0) 9 (36.0) 7 (28.0)	10 (33.3) 12 (40.0) 8 (26.7)	Fisher’s exact test	0.704
**Age at valve ablation** ***(N)***	13	35	48		
Median (IQR) mo	50.7 (29.3–134.0)	26.1 (12.8–45.5)	30.3 (12.8–54.8)	Rank-sum test	0.063

CKD: chronic kidney disease; DMSA Scan: ^99m^Tc dimercaptosuccinic acid renal scan; VUR: vesicoureteral reflux

During our follow up we found 19, 14, 11, 7 and 4 boys with failure to thrive, proteinuria, bladder dysfunction, hypertension and hematuria, respectively. Hypertension, failure to thrive, acute kidney injury and hematuria were significantly higher in the CDK boys.

## Discussion

From our 77 PUV boys, a plurality were diagnosed at 0–3 months of age and over half before 6 months. There were no prenatal diagnoses, and about 10% were diagnosed after 5 years. This age-of-diagnosis breakdown is similar to a study in 65 Thai boys from Bangkok in which the median age at diagnosis was 0.5 years (IQR 0.3–1.3) [21] while the median age at diagnosis in a study of 18 Korean boys was 22.0 months [4]; interestingly, in a study in 98 Iranian boys, 67% were diagnosed before one month of age and 20% were prenatally diagnosed [22].

The number of PUV boys was highest in the middle decade of our study, the 2000s, which is likely related to the fact that our institution was only established in 1982, and for the first decade we were just getting established in a low-resource developing country, but by the 2000s, the middle decade of the study, we were becoming well-established as the major referral center in southern Thailand and increasingly more cases were being referred to us, but then in the 2010s, the number of cases began to decrease as more specialists were working in the provincial and local hospitals in our region. Also in recent years, our hospital expansion has been limited by the number of beds, and only severe referral cases have been accepted (as indicated by the number of cases in our analysis which were returned to their original institution or lost to follow up due to missing medical data more than one year after the last visit).

The most common presentation of PUV is symptoms/signs of urine retention (poor urine steam, dribbling) and related complications (UTI, AKI). In severe PUV, the patient may present with urine ascites, urinoma, palpable, firm urinary bladder, AKI, renal dysplasia, and/or CKD [6, 9, 21, 23]. UTI is the most common sign indicating congenital anomalies of the kidney and urinary tract (CAKUT), and earlier studies reported that around 30% of CAKUT patients had UTI as the first sign [24, 25]. In our study, UTI was the most common presentation, although the previously noted study in Bangkok boys reported that 81% of their patients presented with UTI [21]. UTI is the most frequent morbidity in PUV, and in our study, three-fourths of the boys were complicated with UTI, and two-thirds had recurrence.

In our study, renal ultrasounds revealed a significant number of severe grades of hydronephrosis and/or bilateral hydronephrosis as well as VURs which indicated lower bladder obstruction, resulting in high pressure backing up through the ureter and renal pelvis which can cause renal damage. Severity of hydronephrosis, VUR and renal damage were not significantly different between the CKD and non-CKD boys at their last visits.

Urinary dribbling and poor urine stream are definitive symptoms of bladder obstruction, but were found in only 24% and 14% of our boys, respectively, while studies from Iran and Nigeria found poor urine stream in 43% [11] and 91% [23], respectively. Our low numbers could be explained by noting that urinary symptoms in infants may not be noticed by parents for two reasons, first, the infant is wearing a diaper, or second, was born with the symptoms and the parents, having seen only these voiding manifestations in the infant, think they are normal.

Renal failure, the most serious complication of PUV, was found in 8 boys at the first visit, particularly in the 0–3 months group. In these 8 boys, renal function returned to normal in 6 boys after urinary drainage, while impaired renal function persisted in 2 boys. We did not find AKI in the older boys, as their initial PUV had been diagnosed and corrected at their first visit to their local hospitals, and they were only referred to us later for management of other PUV consequences following the AKI resolution.

Kidney function deterioration develops in utero and continues even after valve ablation since bladder dysfunction remains and causes pressure back to the kidney leading to various possible complications, including recurrent UTI.

Diagnosis at a younger age may result from a patient having severe symptoms of PUV and thus the parents seeking medical attention. At the end of our study, the median age of the boys was 6.3 years, and one-fourth of them had developed CKD. Caione et al. [10] followed 24 patients in Italy aged 18–34 years (mean 23) for 16–30 years (mean 19.5), of whom 13 (54.1%) and 5 (20.8%) had CKD and ESRD, respectively. Although the prevalence of CKD in PUV in our study was not comparable to the Caione study, due to different durations of follow up, the natural course of CKD is that the longer the follow up period, the higher the chance of CKD. Additionally, age at CKD diagnosis may have been delayed in some of our cases, since we are a referral center, and when some of the patients were brought to us for their first visit, the CKD was already advanced, and thus our data on the age of CKD diagnosis may have been incorrect, reflecting an older-than-actual age of CKD development, further resulting in a higher-than-normal probability of renal survival.

Usually, early diagnosis of PUV leads to early treatment and thus better results than in cases of delayed diagnosis. However, early diagnosis may also occur in cases with severe symptoms and/or complications leading to medical investigations, in which case the outcomes may be poor. If possible, intrauterine diagnosis and fetal intervention to decompress the urinary bladder are the best modalities to reduce further renal damage and allow normal lung development [24]. In our study, the average age of the boys who had CKD at their last visit was significantly higher than the boys who did not have CKD (11.9 and 4.6 years, respectively), which reflects the fact that kidney function deteriorates over time, as further evidenced by the fact that the 10-year renal survival was 73.3%, also suggesting that the non-CKD boys were too young to have had CKD development.

Valve ablation is the definitive PUV correction, however, this procedure is usually delayed due to endoscopic size limitations, or a surgeon who can do the procedure is not available; urinary diversion should be done immediately after diagnosis, with initial tube insertion into the bladder for urinary drainage followed by vesicostomy or cystostomy [22]. Our boys had valve ablation at the median age of 2.5 years, which is quite late compared to the report from Bangkok in which their boys had valve ablation at the median age of 0.75 years [21].

Although PUV is a correctable congenital anomaly, damage may occur before surgical correction. The earliest possible post-natal urinary drainage is desirable, but, unfortunately, in low-resource countries many prenatal modalities are not available, including neonatal cystoscopy.

In high-resource countries, modern advances in technology and equipment enable early diagnosis and treatment, leading to much better outcomes than in earlier times. But if a PUV causes renal damage in utero, the long-term outcomes remain unfavorable [7, 24]. After surgical correction, kidney and urinary bladder functions still need lifelong monitoring for delayed renal deterioration to maintain the patient’s quality of life.

Bladder dysfunction often persists even after complete valve ablation [11]. Surprisingly, one study found that bladder dysfunction was not a concern of a majority of parents, so there were few follow ups or urodynamic studies [25]. This was also a limitation of our study, since during our follow up period, 11 boys developed bladder dysfunction, but this could be an underestimation since the diagnosis of this condition was usually based on a patient’s history without urodynamic studies.

Failure to thrive, hypertension and proteinuria are the most common results of CKD, and in our study were found significantly more in the CKD group than in the group in which CKD had not yet developed. Failure to thrive is one of the most common consequences of CKD, however this may also be a consequence of chronic infection since PUV patients tend to have frequent recurrences of UTI. The prevalence of failure to thrive was also influenced by the duration of the follow up period. During the follow up period in our study, 25% of the boys showed signs of failure to thrive, compared to 15% of the Thai boys from the Bangkok study mentioned previously, even though the mean follow-up period of their study was 7.5 years and ours was 6.3 years.

## Conclusion

77 PUV Thai boys over a 30-year period had an average 10-year renal survival of 73%. UTI was the most common presentation. Recurrent UTI was the most frequent consequence, followed by urinary bladder dysfunction. The PUV in our boys was also a severe CAKUT, a disease which develops initially in utero and has high morbidity and high mortality, and all of these patients were scheduled for lifelong follow up for renal and bladder functions.

## Data Availability

The datasets used and/or analysed during the current study are available from the corresponding author on reasonable request.

## Abbreviations

AKI: acute kidney injury
CAKUT: congenital anomalies of the kidney and urinary tract
CKD: chronic kidney disease
DMSA: ^99m^Tc dimercaptosuccinic acid
ESRD: end-stage renal disease
PUV: posterior urethral valve
UTI: urinary tract infection
VUR: vesicoureteral reflux
